# Irish general practitioner attitudes toward decriminalisation and medical use of cannabis: results from a national survey

**DOI:** 10.1186/s12954-016-0129-7

**Published:** 2017-01-13

**Authors:** Des Crowley, Claire Collins, Ide Delargy, Eamon Laird, Marie Claire Van Hout

**Affiliations:** 1 School of Health Sciences, Waterford Institute of Technology, Waterford, Ireland; 2 Irish College of General Practitioners, Dublin, Ireland; 3 School of Biochemistry and Immunology, Trinity College Dublin, Dublin, Ireland

**Keywords:** Cannabis, Decriminalisation, Legalisation, Medical use, Cannabis for therapeutic purposes (CTP), General practitioner

## Abstract

**Background:**

Governmental debate in Ireland on the de facto decriminalisation of cannabis and legalisation for medical use is ongoing. A cannabis-based medicinal product (Sativex®) has recently been granted market authorisation in Ireland. This unique study aimed to investigate Irish general practitioner (GP) attitudes toward decriminalisation of cannabis and assess levels of support for use of cannabis for therapeutic purposes (CTP).

**Methods:**

General practitioners in the Irish College of General Practitioner (ICGP) database were invited to complete an online survey. Anonymous data yielded descriptive statistics (frequencies, percentages) to summarise participant demographic information and agreement with attitudinal statements. Chi-square tests and multi-nominal logistic regression were included.

**Results:**

The response rate was 15% (*n* = 565) which is similar to other Irish national GP attitudinal surveys. Over half of Irish GPs did not support the decriminalisation of cannabis (56.8%). In terms of gender, a significantly higher proportion of males compared with females (40.6 vs. 15%; *p* < 0.0001) agreed or strongly agreed with this drug policy approach. A higher percentage of GPs with advanced addiction specialist training (level 2) agreed/strongly agreed that cannabis should be decriminalised (54.1 vs. 31.5%; *p* = 0.021). Over 80% of both genders supported the view that cannabis use has a significant effect on patients’ mental health and increases the risk of schizophrenia (77.3%). Over half of Irish GPs supported the legalisation of cannabis for medical use (58.6%). A higher percentage of those who were level 1-trained (trained in addiction treatment but not to an advanced level) agreed/strongly agreed cannabis should be legalised for medical use (*p* = 0.003). Over 60% agreed that cannabis can have a role in palliative care, pain management and treatment of multiple sclerosis (MS). In the regression response predicator analysis, females were 66.2% less likely to agree that cannabis should be decriminalised, 42.5% less likely to agree that cannabis should be legalised for medical use and 59.8 and 37.6% less likely to agree that cannabis has a role in palliative care and in the treatment of multiple sclerosis (respectively) than males.

**Conclusions:**

The majority of Irish GPs do not support the present Irish governmental drug policy of decriminalisation of cannabis but do support the legalisation of cannabis for therapeutic purposes. Male GPs and those with higher levels of addiction training are more likely to support a more liberal drug policy approach to cannabis for personal use. A clear majority of GPs expressed significant concerns regarding both the mental and physical health risks of cannabis use. Ongoing research into the health and other effects of drug policy changes on cannabis use is required.

## Background

Cannabis is the most prevalent illicit drug used globally [1]. The policy landscape around cannabis is dynamic [2], with three drug policy options available, prohibition, decriminalisation and legalisation [3]. In recent years, the legal status of cannabis has been extensively debated in academic and policy domains, with many jurisdictions making or considering major changes in their domestic laws and policies on this issue. The International Centre for Science in Drug Policy have advised that such policy responses need to be based on best available evidence [4].

The United Nations (UN) Drug Conventions support the prohibitionist approach and require signatories to make the possession of cannabis a criminal offence under their domestic laws [5]. Policy makers challenging this approach argue that the criminalisation of cannabis is an expensive and ineffective drug control policy which has no impact on reducing availability or use despite its significant financial costs [6]. Proponents for policy change support a less punitive approach to cannabis users with a greater emphasis on public health and human rights [2, 5, 6]. The decriminalisation drug policy approach involves the removal of criminal sanctions for those found in possession of small amounts of the drug for their own use [5]. However, the use of the drug is not legal, and non-criminal offences may still be applied [5]. Underpinning this approach is the argument that cannabis is a relatively safe drug when compared to alcohol and tobacco, and criminal sanctions for personal use and possession of small amounts are excessive [6]. Prohibitionist continues to argue that removal of criminal barriers will lead to increased supply, use, dependence and harm [3].

With increasing public support for the decriminalisation of cannabis use, some jurisdictions are now developing and implementing a range of legally regulated market models for recreational cannabis use [2]. Legalisation of cannabis usually refers to the removal of all criminal and non-criminal sanctions for its use, production and supply. However, legal regulation will set out the rules and restrictions around how these can occur [5].

Despite widespread prohibition, many multiple sclerosis and chronic pain patients use cannabis for analgesia and psychological support [7]. Some states in the USA have moved toward allowing individuals to use cannabis for medical purposes [5], with resultant debate on whether these laws have led to increased use [8]. Debates around legalisation of medical cannabis centre on the beliefs that cannabis has medical effects, medical cannabis is addictive and that legalisation leads to increased use for recreation (i.e. spillover effects) [9]. Some reports indicate the potential value of cannabis for therapeutic purposes (CTP) [10–15]. However, some concerns around the addictive potential of cannabis are evident [4, 5, 16] but with a weak evidence base indicating lower probability of dependence and physical and social harm when compared to alcohol, cocaine, opiates and nicotine [4]. Legalisation in the form of de facto legal supply for medical use (CTP) does not appear to have created a significant threat to public health and safety, or caused increased consumption or related harms [17–20]. In terms of patient experience, legalisation appears to increase patient’s feelings of safety and awareness [21].

To date, eight US states (Alaska, California, Colorado, Oregon, Massachusetts, Maine, Nevada and Washington) have now voted to legalise cannabis for both medical and non-medical use, and one state (Washington DC) has legalised it for personal use but not commercial sale [5]. Regulatory debates are ongoing in many countries including Canada, Jamaica, Italy, Spain, several Latin American countries and additional US states [5].

To date, research on medical practitioner views on this issue remains scant [17]. In the past 20 years, only a minority of oncologists surveyed in the USA were in support of the rescheduling of cannabis for medical purposes [22] and the availability of cannabis on prescription [23]. More recently, in the USA, medical practitioner views on medical cannabis are generally less supportive of medical use, underpinned by recognition of its potential for serious mental and physical health risks [24, 25]. Israeli practitioners have voiced partial acceptance for therapeutic use, with further research warranted to investigate educational and regulatory needs [26]. Israeli rheumatologists in 2016 report majority opinion of some role for cannabinoids in the management of rheumatoid disease [27].

Our study is the first of its kind in Ireland and aimed to investigate Irish general practitioner (GP) attitudes toward decriminalisation of cannabis and assess levels of support for CTP. The study was undertaken on foot of several regulatory developments and debates in Ireland, in particular, a recent governmental debate and reporting from the Oireachtas Justice Committee on the proposed decriminalisation of cannabis. The committee recommended that possession of small amounts of cannabis for personal use should not be dealt with through the criminal justice system and that small-scale users should be treated with compassion. Other debates centred on CTP in Ireland with authorisation necessary from the Health Products Regulatory Authority (HPRA) or, in the case of certain medicinal products, the European Medicines Agency (EMA). In July 2014, regulations were amended to allow for certain cannabis-based medicinal products to be used in Ireland. The HPRA granted a marketing authorisation for a cannabis-based medicinal product (Sativex®) which is indicated for the treatment of spasticity for people with multiple sclerosis. However, the most recent national treatment data indicates cannabis is the most common problem drug among new cases presenting for drug treatment [28].

## Methods

In Ireland, medical training for general practitioners is overseen by the Irish College of General Practitioners (ICGP). Training in substance misuse is provided as levels 1 and 2 training. GPs trained to these levels have acquired additional knowledge and experience in the treatment and management of substance use problems. Ethical approval was granted by the ICGP. In early 2016, an online survey was undertaken with all GPs in the ICGP database based in the Republic of Ireland. Electronic invitations included information on the study and consent information, and a link to access the online survey was sent. A notice encouraging response was also placed on the substance misuse section of the ICGP website. The survey was designed based on a review of the literature and in consultation with the project team. The instrument contained a series of closed questions relating to participant profile and practice location, specialist registration, experience in treating opioid users and their responses toward a five-point Likert scale measuring agreement with a series of statements. Statements measured attitude toward decriminalisation of cannabis, legalisation for CTP, potential for decriminalisation to increase cannabis use, adverse mental and physical health effects of cannabis use, cannabis use in young people and risk of development of schizophrenia and role of CTP in pain management, treatment of multiple sclerosis and palliative care. The survey took an estimated 10 min to complete. While the response rate for this online survey was low (15%), it is consistent with the response rates from GPs nationally and internationally for similar type of studies [29–31].

Anonymous data yielded descriptive statistics (frequencies, percentages) to summarise participant demographic information and agreement with attitudinal statements. Statistical analysis was performed using the Statistical Package for Social Sciences (Version 21.0; SPSS UK Ltd; Chersey, UK). Where appropriate, chi-square tests were applied to determine the statistical significance (*p* < 0.05). Multi-nominal logistic regression analysis was used to determine predictors of answer responses with the neutral response category as the reference.

## Results

### Participant characteristics

The participant characteristics are displayed in Table 1. There was no significant difference in the proportion of males or females sampled (*p* = 0.180). The majority of males (50.5%) tended to be older (>50 years), while the majority of females were aged between 30–50 years (62.4%). Most of the participants were full-time practitioners, (71.3%) though significantly more males were retired compared with females (8.6 vs. 4.2%; *p* = 0.030), while significantly more females were trainees (23.0 vs. 15.1%; *p* = 0.017). Over 70% of respondents were on the GP Specialist Register of the Irish Medical Council with 85% working in general practice. Nearly 30% of the sample was level 1-trained managing opioid users, while only 4.4% were level 2-trained. There was no difference across gender in the practice population with the majority working in an urban area (43.7%) with nearly 40% working in an area of deprivation. Selection bias is limited as all demographics of the responding group are consistent with the national data on GPs [32, 33] (Table 1).

**Table 1:** Participant characteristics

	Total (*n* 565)	Male (*n* 278)	Female (*n* 287)	*p* value
Age *n* (%)				
<30 years	41 (7.3)	16 (5.8)	25 (8.7)	0.180
30–50 years	300 (53.2)	121 (43.7)	179 (62.4)	0.288
>50 years	223 (39.5)	140 (50.5)	83 (28.9)	<0.0001
Membership of the ICGP *n* (%)				
Associate, part-time, others	18 (3.2)	8 (2.9)	10 (3.5)	0.681
Full-time	403 (71.3)	204 (73.4)	199 (69.3)	0.288
Retired	36 (6.4)	24 (8.6)	12 (4.2)	0.030
Trainee	108 (19.1)	42 (15.1)	66 (23.0)	0.017
Training *n* (%)				
On the GP specialist register	435 (77.5)	216 (78.3)	219 (76.8)	0.687
Working in general practice	482 (85.3)	242 (87.1)	240 (83.6)	0.250
Working in academic general practice	129 (23.0)	64 (23.4)	65 (22.7)	0.859
Level 1-trained GP managing opioid users	169 (29.9)	77 (27.7)	92 (32.1)	0.258
Level 2-trained GP managing opioid users	25 (4.4)	16 (5.8)	9 (3.1)	0.130
Practice population *n* (%)				
Mixed	211 (39.7)	110 (41.5)	101 (38.0)	0.405
Rural	88 (16.6)	42 (15.8)	46 (17.3)	0.655
Urban	232 (43.7)	113 (42.6)	119 (44.7)	0.626
Working in an area of deprivation	211 (39.4)	99 (37.4)	112 (41.5)	0.329

Values are *n* (%); chi-square analysis for categorical variables for comparisons of distributions between gender

### Statement responses

The answer responses were examined by gender, age and training level. A significantly higher proportion of males compared with females (40.6 vs. 15.0%; *p* < 0.0001) agreed or strongly agreed that cannabis should be decriminalised, while more males than females also agreed that cannabis should be legalised for medical use (*p* = 0.002). There were no significant differences in the responses to the remaining questions by gender; for example, approximately 80% of both genders agreed that cannabis use has a significant effect on patients’ mental health and increases the risk of schizophrenia (77.3%), while over 60% of both males and females agreed that cannabis can have a role in pain management, multiple sclerosis and palliative care (Table 2).

**Table 2:** Statement responses by gender

	Total	*n* (%) Agreed		
		Female	Male	*p* value
Cannabis should be decriminalised	156 (27.6)	43 (15.0)	113 (40.6)	<0.0001
Cannabis should be legalised for medical use	331 (58.6)	146 (50.9)	185 (66.5)	0.002
The decriminalisation of cannabis use would lead to its increased use	373 (66.0)	200 (69.7)	173 (62.2)	0.089
Cannabis use has a significant adverse effect on patients’ mental health	467 (82.7)	246 (85.7)	221 (79.5)	0.346
Cannabis use has a significant adverse effect on patients’ physical health	339 (60.0)	175 (61.0)	164 (59.0)	0.111
Cannabis use among young people increases the risk of schizophrenia	437 (77.3)	228 (79.4)	209 (75.2)	0.990
Cannabis has a role to play in pain management	359 (63.5)	166 (57.8)	193 (69.4)	0.511
Cannabis can have a role in the treatment of multiple sclerosis	352 (62.3)	165 (57.5)	187 (67.3)	0.914
Cannabis can have a role in palliative care	387 (68.5)	175 (61.0)	212 (76.3)	0.362

Total agreed (strongly agree and agree). Values are *n* (%); chi-square analysis for categorical variables for comparisons of distributions between gender

When analysed by age, only 29% of those aged <50 years agreed that cannabis should be decriminalised. In contrast, a significantly higher proportion of those aged >50 years agreed that cannabis should be legalised for medical use compared to the younger GPs sampled (71.1 vs. 79.6%; *p* = 0.044). When analysed by level of training, a higher percentage of those who were level 1-trained agreed/strongly agreed cannabis should be legalised for medical use (*p* = 0.003). However, a significantly lower proportion of this group agreed/strongly agreed that cannabis has a role in pain management (*p* = 0.032), in treatment of multiple sclerosis (*p* = 0.037) and in palliative care (*p* = 0.047) compared to those who are not level 1-trained. In contrast, a higher percentage of those level 2-trained agreed/strongly agreed that cannabis should be decriminalised compared to those without this training (54.1 vs. 31.5%; *p* = 0.021) (Tables 3, 4 and 5).

**Table 3:** Statement responses by age

	*n* (%) Agreed		
	<50 years	>50 years	*p* value
Cannabis should be decriminalised	85 (29.7)	71 (37.3)	0.082
Cannabis should be legalised for medical use	190 (71.1)	141 (79.6)	0.044
The decriminalisation of cannabis use would lead to its increased use	232 (82.2)	141 (82.4)	0.960
Cannabis use has a significant adverse effect on patients’ mental health	281 (92.4)	185 (92.5)	0.978
Cannabis use has a significant adverse effect on patients’ physical health	210 (81.3)	128 (81.5)	0.973
Cannabis use among young people increases the risk of schizophrenia	269 (91.8)	167 (89.3)	0.354
Cannabis has a role to play in pain management	221 (88.4)	138 (82.1)	0.072
Cannabis can have a role in the treatment of multiple sclerosis	208 (92.4)	144 (87.8)	0.123
Cannabis can have a role in palliative care	235 (90.0)	152 (85.3)	0.139

Total agreed (strongly agree and agree). Values are *n* (%); chi-square analysis for categorical variables for comparisons of distributions between age

**Table 4:** Statement responses by training level (level 1)

	*n* (%) Agreed		
	Not level 1	Level 1	*p* value
Cannabis should be decriminalised	114 (34.7)	42 (28.1)	0.156
Cannabis should be legalised for medical use	245 (78.5)	86 (65.1)	0.003
The decriminalisation of cannabis use would lead to its increased use	264 (83.0)	109 (80.1)	0.464
Cannabis use has a significant adverse effect on patients’ mental health	321 (92.2)	146 (92.9)	0.767
Cannabis use has a significant adverse effect on patients’ physical health	229 (80.3)	110 (83.9)	0.377
Cannabis use among young people increases the risk of schizophrenia	301 (89.5)	136 (93.7)	0.142
Cannabis has a role to play in pain management	262 (88.2)	97 (80.1)	0.032
Cannabis can have a role in the treatment of multiple sclerosis	257 (92.4)	95 (85.5)	0.037
Cannabis can have a role in palliative care	282 (90.0)	105 (83.3)	0.047

Total agreed (strongly agree and agree).Values are *n* (%); chi-square analysis for categorical variables for comparisons of distributions between level 1 training

**Table 5:** Statement responses by training level (level 2)

	*n* (%) Agreed		
	Not level 2	Level 2	*p* value
Cannabis should be decriminalised	143 (31.5)	13 (54.1)	0.021
Cannabis should be legalised for medical use	313 (74.1)	18 (81.8)	0.422
The decriminalisation of cannabis use would lead to its increased use	360 (82.7)	13 (68.4)	0.110
Cannabis use has a significant adverse effect on patients’ mental health	446 (92.7)	21 (87.5)	0.344
Cannabis use has a significant adverse effect on patients’ physical health	323 (81.7)	16 (76.1)	0.521
Cannabis use among young people increases the risk of schizophrenia	416 (90.6)	21 (95.4)	0.443
Cannabis has a role to play in pain management	341 (85.6)	18 (90.0)	0.588
Cannabis can have a role in the treatment of multiple sclerosis	337 (90.3)	15 (93.7)	0.650
Cannabis can have a role in palliative care	369 (88.2)	18 (85.7)	0.723

Total agreed (strongly agree and agree). Values are *n* (%); Chi-square analysis for categorical variables for comparisons of distributions between level 2 training

In the regression response predicator analysis, females were 66.2% less likely to agree that cannabis should be decriminalised, and 42.5% less likely to agree that cannabis should be legalised for medical use compared with males. Females were also 59.8 and 37.6% less likely to agree that cannabis has a role in palliative care and a role in the treatment of multiple sclerosis (respectively) than males. Furthermore, GPs based in a non-deprived area were 56.4% more likely to agree that cannabis has a role in pain management compared to those working in a deprived area, while GPs aged 30–50 years were 189.5% more likely to disagree that cannabis can have a role in the treatment of multiple sclerosis compared to those not in that age group (Table 6).

**Table 6:** Statement response predicators

	Category	Variable	Beta	Exp (B)	*p* value	95% CI	
						lower	upper
Cannabis should be decriminalised	Agree	Female	−1.084	0.338	<0.0001	0.19	0.602
Cannabis should be legalised for medical use	Agree	Female	−0.553	0.575	0.017	0.365	0.907
Cannabis has a role to play in pain management	Agree	Female	−0.644	0.525	0.003	0.343	0.805
	Agree	Non-deprived area	0.447	1.564	0.041	1.018	2.403
	Disagree	Not level 1	−0.706	0.494	0.049	0.244	0.998
Cannabis can have a role in the treatment of multiple sclerosis	Agree	Female	−0.472	0.624	0.019	0.42	0.926
	Disagree	Aged 30–50 years	1.063	2.895	0.013	1.257	6.668
Cannabis can have a role in palliative care	Agree	Female	−0.91	0.402	<0.0001	0.255	0.636

Multi-nominal logistic regression analysis; reference category is neutral response

## Discussion

This study yielded unique findings with regard to Irish GP views on decriminalisation of cannabis and the medical use of cannabis (CTP). The majority of GPs did not support the decriminalisation of cannabis, but male respondents were significantly more likely to support this drug policy approach than their female counterparts. This majority view is contrary to current Irish government policy on the issue. Nearly 60% of GPs supported the legalisation of cannabis for medical use with older (>50) and male GPs more likely to favour this approach. In contrast to the view on decriminalisation, this majority view supports Irish government policy on CTP.

In 2000, the Irish *Drug Related Knowledge, Attitudes and Beliefs* survey found that males reported greater use of cannabis and knowledge of cannabis users than females [34]. This study, while dated, indicated that only 24% were in favour of legalisation of cannabis. Of interest is that the majority of GPs trained to a more specialist level in addictions supported decriminalisation (54.1% *p* = 0.021). This may in part be due to their experience treating patients with opioid dependence in particular injecting drug users. In this patient cohort, cannabis use is often considered a secondary issue when managing the high-risk behaviour and health issues associated with injecting drug use. In contrast, in terms of gender, regression analyses revealed that females were less likely to agree that cannabis should be decriminalised. Prevalence data in Ireland has reported little change in women’s rates of cannabis use, with prevalence rates highest among men and younger adults aged 15–34 years [35]. Research shows that individuals with personal experience of cannabis use are more inclined to be in favour of legalising [36]. According to the National Advisory Committee on Drugs and Alcohol (NACDA) in 2012 general population survey, the majority of respondents agreed with medical use of cannabis and disagreed with recreational use of cannabis. This is consistent with the drug policy approaches supported by Irish GPs in this survey.

Both genders of GPs in this study voiced concerns that decriminalisation would lead to increased use and the misuse of prescribed versions if available. Hall and Lynskey [37] speculate that potential effects of legalising recreational cannabis use, while substantially reducing the price, increasing excessive use and related harms, may also increase the number of new users. However, of interest, is recent reporting of a great overlap between medicinal and recreational cannabis use in the USA [38]. Regulating the cannabis market via prohibition and decriminalisation has a marginal effect on onset of cannabis use and population consumption rates [6]. Yuyan et al. [39] in their review of 38 countries indicated that cannabis liberalisation with depenalization and partial prohibition policies was associated with higher levels of regular cannabis use among adolescents. This is concerning given a recent Irish study [40] which underscored the relatively low levels of adolescent-perceived risk of mental and physical health problems with use of the drug. According to Eurobarometer [41], Ireland has the highest number of young people who have used cannabis in the past year (28%), compared to a Europian Union (EU) average of 17%; 58% of Irish youth have never taken cannabis, which in 2014 is third lowest in Europe and well below the EU average of 69%. Overall, 46% of young Irish people consider regular cannabis use to be high risk, compared to an EU average of 63%. The Eurobarometer in 2014 [41] also reported that 56% of Irish 15–24-year-olds were of the view that the cannabis market should be regulated, which is almost a reverse position of the EU average, where 45% said cannabis should be regulated, and 53% said it should continue to be banned.

GPs in this national study agreed that cannabis use has a significant effect on patients’ mental health and increases the risk of schizophrenia. Kondrad and Reid [25] have reported majority agreement of US physicians in Colorado regarding the serious mental and physical health risks of cannabis. Irish GPs in this study underscored their experience of the significant adverse effect on patient mental health and risks of schizophrenia in young users. Heavy use of cannabis at an earlier age is reported to heighten risk of dependence, depressive disorders, psychosis, suicidal ideation, development of schizophrenia and related negative social consequences [17]. This contributes to the debate around the cannabinoid hypothesis of psychosis, for example that exposure to cannabis and cannabinoid agonists is associated with psychosis outcomes through activation of CB1R [42–44]. However, to date, a causal relationship has not been established [4].

Kondrad and Reid [25] in their 2013 study of physician experiences and attitudes to medical cannabis reported that nearly half of participants did not support CTP and that only a minority thought therapeutic use of cannabis conferred physical and mental health benefits. Israeli practitioners in 2015 voiced partial acceptance for therapeutic use, and generally agreed that CTP could be helpful for chronic and for terminally ill patients [26]. However, oncologists and pain specialists did not agree unanimously that medical cannabis can undermine mental health, whereas other physicians did [26]. CTP is driven by public approval without scientific data routinely required to justify new medication regulation [45, 46]. In 2005, US physicians reported less support of CTP than the public [24]. Irish GPs who reported practicing in a non-deprived area in this study were more likely to agree that cannabis has a role in pain management compared to those who reported working in a deprived area. Level of training for this Irish sample reflected that a greater percentage of those participants with level 1 substance misuse training agreed/strongly agreed that cannabis should be legalised for medical use. While the clear majority of this group supported the role of medical cannabis in pain management, treatment of multiple sclerosis and palliative care, they were statistically less supportive than those without this training. Irish GPs aged 30–50 years were more likely to disagree that cannabis can have a role in the treatment of multiple sclerosis compared to those not in that age group. Kondrad and Reid [25] found that age was significantly associated with the recommendation of medical cannabis. They also found no significant association with physician gender, years in practice, or type of licence and recommendation of medical cannabis. In contrast, this study found that female GPs in Ireland were less likely to agree that cannabis should be legalised for medical use compared with males, and were also less likely to agree that cannabis has a role in palliative care or a role in the treatment of multiple sclerosis than males.

## Conclusions

The study is unique in terms of describing Irish GP views on different drug policy approaches to cannabis use and is only one of a handful of studies worldwide that examines physicians’ views on this topical issue. The majority of Irish GPs do not support decriminalisation of cannabis use which is now the policy choice of the Irish government. This study found interesting differences in views between male and female GPs and those with different levels of addiction training and experience. Female GPs tended to favour a more conservative drug policy approach, while those with more advanced training supported a more liberal one. The finding of majority support for CTP among Irish GPs in this study underpins and supports the proposed legislative changes that are presently being considered by the Irish parliament. While supporting this policy initiative, a clear majority of GPs viewed that such an approach had the potential to increase cannabis use and expressed concerns regarding both the mental and physical health risks associated with its use. This study captures the views of Irish GPs on potential policy changes regarding both medical and recreational cannabis use at a time when both are prohibited and will facilitate further research on how these views may change over time and be influenced by a different policy approach. Further research is warranted to explore how gender and levels of addiction training and experience impact doctors’ views on drug policy.

## Abbreviations

CTP: Cannabis for therapeutic purposes
EMA: European Medicines Agency
EU: European Union
GP: General practitioner
HPRA: Health Products Regulatory Authority
ICGP: Irish College of General Practitioners
NACDA: National Advisory Committee on Drugs and Alcohol
UN: United Nations
